# Exposure Time Optimization for Highly Dynamic Star Trackers

**DOI:** 10.3390/s140304914

**Published:** 2014-03-11

**Authors:** Xinguo Wei, Wei Tan, Jian Li, Guangjun Zhang

**Affiliations:** Key Laboratory of Precision Opto-mechatronics Technology, Ministry of Education, School of Instrumentation Science and Opto-electronics Engineering, Beihang University, Beijing 100191, China; E-Mails: tanweibuaa@163.com (W.T.); lijian_0355@163.com (J.L.)

**Keywords:** star tracker, highly dynamic, exposure time, star detection sensitivity, star location error

## Abstract

Under highly dynamic conditions, the star-spots on the image sensor of a star tracker move across many pixels during the exposure time, which will reduce star detection sensitivity and increase star location errors. However, this kind of effect can be compensated well by setting an appropriate exposure time. This paper focuses on how exposure time affects the star tracker under highly dynamic conditions and how to determine the most appropriate exposure time for this case. Firstly, the effect of exposure time on star detection sensitivity is analyzed by establishing the dynamic star-spot imaging model. Then the star location error is deduced based on the error analysis of the sub-pixel centroiding algorithm. Combining these analyses, the effect of exposure time on attitude accuracy is finally determined. Some simulations are carried out to validate these effects, and the results show that there are different optimal exposure times for different angular velocities of a star tracker with a given configuration. In addition, the results of night sky experiments using a real star tracker agree with the simulation results. The summarized regularities in this paper should prove helpful in the system design and dynamic performance evaluation of the highly dynamic star trackers.

## 1. Introduction

As the highest accuracy attitude measurement device, star trackers are capable of providing arcsec level 3-axis attitude and are widely used in many spacecrafts. A star tracker can be treated as a special electronic camera connected to a microcomputer. It can take star images of a part of the sky and identify these stars in the star image. Based on the position information of these identified stars, the attitude of spacecraft can be determined [1–4].

With the expansion of the application fields, especially on spacecrafts with the capability of rapid and large angle attitude maneuver control, star trackers must still work normally and steadily. Under these highly dynamic conditions, a crucial problem arises: due to the large angular velocity of the spacecraft, the star-spots in the star image will move across many pixels during the exposure time and ultimately form obvious trails. This will affect star detection sensitivity and star location accuracy seriously and result in low attitude accuracy and poor stability. This case can be ameliorated by adjusting the dynamic working parameters, especially the exposure time. Increasing the exposure time means more energy is gathered at each star-spot, which enhances high star detection sensitivity, but on the other hand, increasing the exposure time aggravates the movement of the star-spots and makes it more difficult to locate them. By contrast, reducing the exposure time alleviates the movement effect and reduces star location errors, but at the cost of an energy loss. Therefore, under highly dynamic conditions there exists an optimal exposure time and it is necessary to choose this proper exposure time for star trackers.

As discussed above, under highly dynamic conditions the exposure time mainly affects two aspects of star trackers: star detection sensitivity and star location accuracy. The exposure time directly determines the total energy and length of each star-spot in the star image, and both of them together affect star detection sensitivity. Star detection sensitivity determines the number of the star-spots, which is an important factor affecting the attitude accuracy and stability of a star tracker. In the past years, some research about star detection sensitivity has been reported. In [5] a rough estimation method for star detection sensitivity utilizing the SNR model for static conditions was first reported. Reference [6] gives a general expression of star detection sensitivity based on the theory described in [5], but under highly dynamic conditions the star-spots model can't use the two-dimensional Gaussian distribution like in static conditions, so that the star detection sensitivity model developed for static conditions is not suitable for dynamic conditions. Reference [7] gives a dynamic star-spot imaging model, and obtains the regularity of star detection sensitivity at different angular velocities for a star tracker.

The movement of the star-spots during the exposure time also increases the difficulty of locating the star-spots and lowers the star location accuracy. The star location accuracy is the primary factor determining the attitude accuracy of a star tracker. In the past, many researchers have concentrated on the exploration of star location errors. Reference [8] obtains the star location error of the ideal star-spots in a 5 × 5 centroiding window by calculation of the effects of various noise components. Reference [9] shows an explicit expression of the S-curve systematic error caused by the different positions of the star-spot center in a certain pixel. Reference [10] gives a typical model of star location error containing a systematic contribution and a random one. However, all these researches mainly focus on static conditions, and we need to make research in depth the star location errors under highly dynamic conditions.

This paper presents a method for optimizing the exposure time from the two aspects: star detection sensitivity and star location accuracy, and obtains the optimal exposure times for different angular velocities of a star tracker. This paper is divided into six sections. Following the Introduction, we first introduce the dynamic star-spot imaging model and star detection sensitivity with regard to the exposure time in Section 2. In Section 3, the star location error is deduced based on the error analysis of the sub-pixel centroiding algorithm, and the effect of the exposure time on the star location error is obtained. Combining the analyses in Sections 2 and 3, the overall effect of exposure time on attitude accuracy is obtained in Section 4, and the optimal exposure time is determined with the highest attitude accuracy as the criterion. Night sky experiments with a real star tracker are carried out in Section 5. Conclusions are drawn in the last section.

## 2. Star Detection Sensitivity

### 2.1. Dynamic Star-Spot Imaging Model

**Figure 1.**
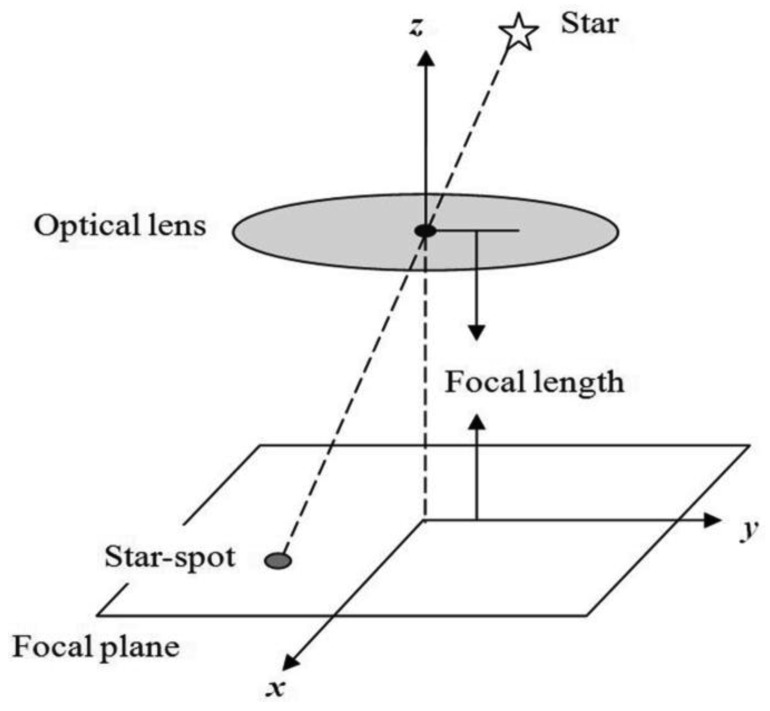
Static star-spot imaging process.

Under static conditions, the angular velocity of the carrier is very low and stars can be assumed to be point sources. The star is projected on the focal plane and this generates a defocused star-spot as shown in Figure 1. The point spread function of the optical system can be described as a two-dimensional Gaussian function, which is the energy distribution shape of the imaged star-spot.

The static star-spot imaging model is given by [11]:

(1) $$S(x,y)=\frac{\mu_{0}}{{2\pi \sigma_{\text{pixel}}}^{2}}\exp\{-\frac{{\left(x-x_{c}\right)}^{2}+{\left(y-y_{c}\right)}^{2}}{{2\sigma_{\text{pixel}}}^{2}}\}$$

where *μ*_0_ is the total energy that star tracker absorbs from a single star during the exposure time, usually expressed using the signal photoelectrons, (*x_c_*, *y_c_*) is the center of the star-spot, and *σ_pixel_* is the Gaussian radius, determined by the defo**c**used extent of the optical system. The total signal photoelectrons *μ*_0_ of the star-spot is calculated using the photoelectrons transmit model [7]:

(2) $$\mu_{0}=\tau_{A}\cdot \tau_{0}\cdot E_{0}\cdot 2.512^{-Mv}\cdot \frac{\pi D^{2}}{4}\cdot T_{e}\cdot QE\cdot \frac{1}{E_{ph}}\cdot K_{fill}$$

where *τ_A_* is the atmospheric transmissivity, *τ*_0_ is the optical transmittance, *E*_0_ = 2.96 × 10^−14^ W/mm^2^, refers to the measured flux (on the Earth in the absence of the atmosphere) of the star with magnitude 0 [12], *Mv* is the magnitude, *D* is the optical aperture, *T_e_* is the exposure time, *QE* is the quantum efficiency of the image sensor, *E_ph_* is the average energy of a single photon, and *K_fill_* is the fill factor of the image sensor.

**Figure 2.**
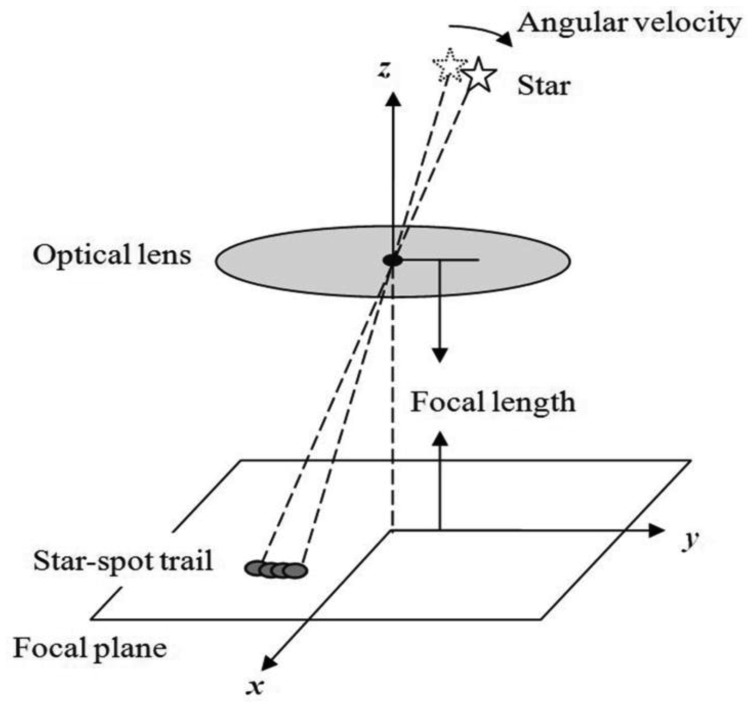
Dynamic star-spot imaging process.

Under highly dynamic conditions, the traditional two-dimensional Gaussian distribution is not suitable because the star-spots obviously move as shown in Figure 2. A tiny moment during the exposure time is taken out and expressed using the *ΔT*(*t*). In this moment the star-spot can be expressed approximately using the traditional two-dimensional Gaussian distribution [7]. Supposing that the star-spot center coordinates at any time *t* in the exposure period is (*x_c_*(*t*), *y_c_*(*t*)) and the total energy during the *ΔT*(*t*) is *μ*_0_(*t*), the star-spot model during this moment can be expressed as:

(3) $$S(x,y,t)=\frac{\mu_{0}(t)}{{2\pi \sigma_{\text{pixel}}}^{2}}\exp\{-\frac{{\left(x-x_{c}(t)\right)}^{2}+{\left(y-y_{c}(t)\right)}^{2}}{{2\sigma_{\text{pixel}}}^{2}}\}$$

Considering the angular velocity of the carrier is basically steady and the exposure time is not long, the star-spot trail can be regarded approximately as a beeline. The length of the star-spot trail can be calculated approximately using *l* = *fwT_e_*/*DX*, where *f* is the focal length, *w* is the angular velocity, *T_e_* is the exposure time and *DX* is the size of each pixel. Defining *θ* as the angle between the star-spot trail and the *x* axis and (*x*_0_, *y*_0_) as the star-spot center coordinates at the time *t* = 0, (*x_c_*(*t*), *y_c_*(*t*)) is approximately:

(4) $$\{\begin{array}{c} x_{c}(t)\approx x_{0}+fwt\cos(\theta )/DX\\ y_{c}(t)\approx y_{0}+fwt\sin(\theta )/DX \end{array}\;(0\leq t\leq T_{e})$$

The total signal photoelectrons in (*x*, *y*) can be obtained by adding up the signal photoelectrons of all moments during the exposure time, so the dynamic star-spot imaging model can be obtained from the integral of *S*(*x*, *y*, *t*):

(5) $$S(x,y)=\frac{\mu_{0}}{{2\pi \sigma_{\text{pixel}}}^{2}T_{e}}\int_{0}^{T_{e}}\exp\{-\frac{{\left(x-x_{c}(t)\right)}^{2}+{\left(y-y_{c}(t)\right)}^{2}}{{2\sigma_{\text{pixel}}}^{2}}\}dt$$

On the real image plane, the total signal photoelectrons of the star-spot are divided into many pixels. The signal photoelectrons of the pixel (*m*, *n*) can be obtained through the integral of the *S*(*x*, *y*) in the pixel position:

(6) $$S(m,n)=\frac{\mu_{0}}{{2\pi \sigma_{\text{pixel}}}^{2}T_{e}}\int_{m-1/2}^{m+1/2}\int_{n-1/2}^{n+1/2}\int_{0}^{T_{e}}\exp\{-\frac{{\left(x-x_{c}(t)\right)}^{2}+{\left(y-y_{c}(t)\right)}^{2}}{{2\sigma_{\text{pixel}}}^{2}}\}\text{dtdydx}$$

### 2.2. SNR Model

Star detection sensitivity *Mv* is a magnitude threshold over which the star can be detected by a star tracker. Under highly dynamic conditions, it can be calculated using the SNR model. The SNR of a pixel is the ratio of its signal photoelectrons to its noise electrons.

**Figure 3.**
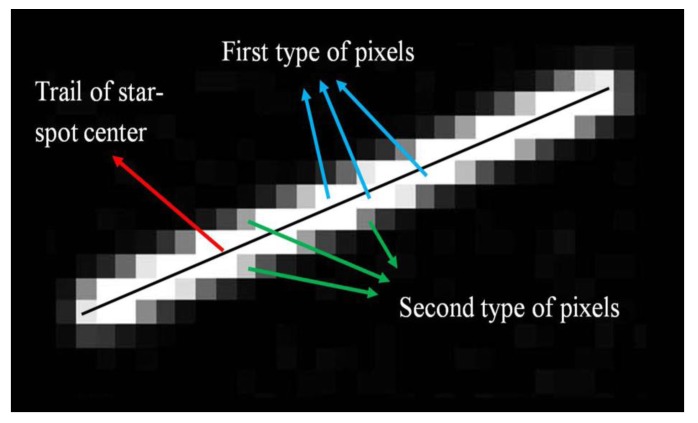
The first and second type of pixels in the star-spot trail.

For analysis convenience, we classify the pixels in the star-spot trail into two types. As shown in Figure 3, the pixels in the middle of the trail are classified as the first type, which includes the most photoelectrons. Those pixels next to the first type belong to the second type, whose photoelectrons are just secondary to the first type of pixels. If these two types of pixels can form a connected domain of a certain size, the star-spot can be correctly extracted from the star image. A criterion can be set that the star can be successfully detected if the second type of pixels in the connected domain meet the SNR requirement [13].

We define *S* as the average signal photoelectrons of the second type of pixels, and *η* as the ratio of the *S* to the total energy *μ*_0_. Taking the CMOS image sensor as an example, the noise electrons *N* of the pixel [14–18] is given as:

(7) $$N=\sqrt{n_{DC}^{2}+n_{S}^{2}+n_{\text{PRNU}}^{2}+n_{TN}^{2}+n_{\text{FPN}}^{2}+n_{\text{ADC}}^{2}}$$

where *n_DC_*, *n_S_*, *n_PRNU_*, *n_TN_*, *n_FPN_*, *n_ADC_* are dark current noise, photon shot noise, photon non-uniformity response noise, temporal noise, fixed pattern noise and analog-to-digital conversion noise, respectively, which are listed in Table 1. Therefore the SNR of the second type of pixels and the star detection sensitivity *Mv* can be expressed as:

(8) $$\text{SNR}=\frac{S}{N}=\frac{\eta \mu_{0}}{\sqrt{n_{DC}^{2}+n_{S}^{2}+n_{\text{PRNU}}^{2}+n_{TN}^{2}+n_{\text{FPN}}^{2}+n_{\text{ADC}}^{2}}}\geq V_{sn}$$

(9) $$Mv\leq -\log_{2.512}(\frac{V_{sn}^{2}+\sqrt{V_{sn}^{4}+4(1-V_{sn}^{2}\sigma_{\text{PRNU}}^{2})V_{sn}^{2}(n_{DC}^{2}+n_{TN}^{2}+n_{\text{FPN}}^{2}+n_{\text{ADC}}^{2})}}{2\eta (1-V_{sn}^{2}\sigma_{\text{PRNU}}^{2})(\tau_{A}\cdot \tau_{0}\cdot E_{0}\cdot \pi D^{2}\cdot T_{e}\cdot QE)/(4\cdot E_{ph})})$$

where *S* is the average signal photoelectrons of the second type of pixels, *N* is the average noise electrons of the same pixels, *V_sn_* is the SNR threshold.

Denoting *A* as the Field Of View (FOV) of the star tracker, for the square shaped CMOS image sensor, the average number of the star-spots *N_star_* in the star image is given by [12]:

(10) $$N_{\text{star}}=6.57\cdot e^{1.08Mv}\cdot \frac{1-\cos(A/\sqrt{\pi })}{2}$$

which indicates the star detection sensitivity *Mv* is the only factor that affects the number of the star-spots in a given FOV.

Simulations are done to obtain the *η* values with different trail lengths (*l* = 0∼19 pixels). The *η* is mainly affected by two factors: the length of the star-spot trail and the angle between the star-spot trail and the *x* axis. The angle depends on the direction of the angular velocity. The results with a 45° angle are shown in Table 2.

**Figure 4.**
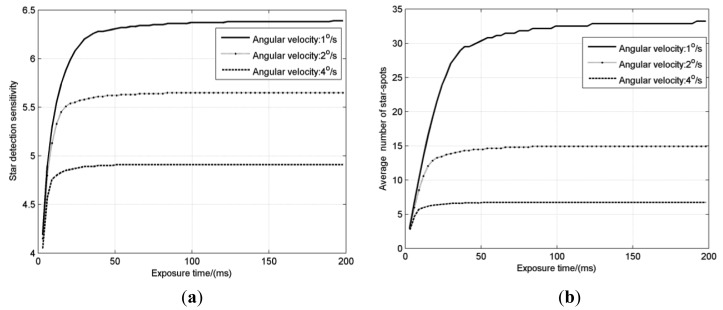
(**a**) Star detection sensitivity *Mv* with different exposure times; (**b**) The average number of star-spots *N_star_* with different exposure times.

The product of the *η* and the trail length *l* approximates to a fixed value 0.264 when the length *l* > 9 pixels, which is a valid generalization that can be used to calculate the *η* when *l* > 19 pixels. The *Mv* and *N_star_* under different exposure times (*T_e_* = 0∼200 ms) and different angular velocities (*w* = 1°/s, 2°/s and 4°/s) are also obtained in Figure 4, with other parameters listed in Table 3.

As shown in Figure 4a, the general regularity is that the star detection sensitivity rises rapidly at first and then slowly approaches a constant with the increasing exposure time. When the exposure time is very short, the star-spot trail is very short, and increasing the exposure time means the gathering of the energy in each pixel, so the SNR and star detection sensitivity rise quickly, but with the continually increase of exposure time, the star-spot moves longer and the total energy spreads out over more pixels. The energy of each pixel begins to increase slowly and finally remains invariable, so that the star detection sensitivity slowly approaches a constant.

The above analysis indicates that star detection sensitivity has a limit value for each angular velocity. In Figure 4a, star detection sensitivity of the star tracker with a given configuration at the angular velocities of 1°/s, 2°/s and 4°/s are 6.38, 5.65 and 4.91, respectively.

In addition, it must be noted that the critical exposure times when the star detection sensitivity approaches the limit value are different for different angular velocities. The higher the angular velocity is, the shorter the critical exposure time is. Under highly dynamic conditions, the increase of exposure time has two effects: the increase of the energy at a single pixel and the dispersion of the energy over several pixels. The critical exposure time, denoted as *T_SDS_*, can be regarded as the balance point of these two effects. In case that the exposure time is over the balance point, increasing the exposure time is useless and cannot produce higher star detection sensitivity.

## 3. Star Location Accuracy

### 3.1. Sub-Pixel Centroiding Algorithm

The sub-pixel centroiding algorithm is utilized to improve star location accuracy. Generally, the optical system of star tracker is slightly defocused, and the star-spots in the star image will occupy several pixels. This will help to calculate the star-spot centroid with sub-pixel accuracy. The sub-pixel centroiding algorithm is given by [19]:

(11) $$\begin{array}{c} \bar{x}=\frac{\iint_{C}xS(x,y)\text{dxdy}}{\iint_{C}S(x,y)\text{dxdy}}=\frac{\int_{0}^{T_{e}}x_{c}(t)dt}{T_{e}}\\ \bar{y}=\frac{\iint_{C}yS(x,y)\text{dxdy}}{\iint_{C}S(x,y)\text{dxdy}}=\frac{\int_{0}^{T_{e}}y_{c}(t)dt}{T_{e}} \end{array}$$

where *C* is a limited area, called centroiding window, *S*(*x*, *y*) is the dynamic star-spot imaging function in Equation (5), and (*x_c_*(*t*), *y_c_*(*t*)) is the star-spot center at the time *t* (0 ⩽ *t* ⩽ *T_e_*).

For the dynamic star-spot, the prescriptive center of the whole star-spot trail is the star-spot center at the time *t* = *T_e_*/2: (*x_c_*(*T_e_*/2), *y_c_*(*T_e_*/2)). According to Equation (4), the *x_c_*(*t*) and *y_c_*(*t*) are approximately linear functions. Consequently, *x̄* ≈ *x_c_*(*T_e_*/2), *ȳ* ≈ *y_c_*(*T_e_*/2), and it is feasible to use the sub-pixel centroiding algorithm to determine the star-spot location, even under highly dynamic conditions. In fact, it must be noted that the available image is discrete, and the imaging function *S*(*x*, *y*) is sampled in each pixel. The real formula used to calculate the star-spot centroid is:

(12) $$\bar{x}=\frac{\overset{n}{\underset{k=1}{\text{∑}}}x_{k}I_{k}}{\overset{n}{\underset{k=1}{\text{∑}}}I_{k}},\bar{y}=\frac{\overset{n}{\underset{k=1}{\text{∑}}}y_{k}I_{k}}{\overset{n}{\underset{k=1}{\text{∑}}}I_{k}}$$

where *n* is the number of pixels in the centroiding window, (*x_k_*, *y_k_*) is the geometric center of the *k*th pixel, and *I_k_* is the sampling value of the *k*th pixel. The sampling value *I_k_* is directly proportional to the amount of signal photoelectrons *S_k_*, which can be calculated using Equation (6):

(13) $$I_{k}=\lambda S_{k},\lambda =\frac{2^{q}}{N_{\text{well}}}$$

where λ is the ratio, *N_well_* is the full well charge of the image sensor, and *q* is the quantization bits. Therefore, according to Equation (7), *I_k_* variance can be expressed as:

(14) $$\sigma_{I_{k}}^{2}=\lambda^{2}N^{2}=\lambda^{2}(n_{DC}^{2}+n_{S,k}^{2}+n_{\text{PRNU},k}^{2}+n_{TN}^{2}+n_{\text{FPN}}^{2}+n_{\text{ADC}}^{2})$$

### 3.2. Star Location Error

Under highly dynamic conditions, star location error of the star tracker using the sub-pixel centroiding algorithm includes the following error sources:

(1) The error from the nonlinearity of the *x_c_*(*t*) and *y_c_*(*t*), which is mainly caused by the variation of the angular velocity [20]. Considering the exposure time is usually very short, the angular velocity during that short time can be approximated as a constant in most cases, so this error is not taken into account in this paper.
(2) The systematic error caused by using the geometric center of the pixel instead of the energy center in the sub-pixel centroiding algorithm. Under static conditions, it changes like the sine curve with the different position of the star-spot center in a pixel, and is usually called as the S-curve error [21].
(3) The random error caused by the random noises of the image sensor, which is determined by the range of the random noises and the length of star-spot trail. Generally, more noises and especially the noises at the pixel farther away from the star-spot centroid will aggravate this kind of error.

For convenience, the following analysis takes the *x̄* as an example. According to Equation (12), the star location error of the *x̄* derives from two sources: *x_k_* and *I_k_*. Assuming that the errors of different pixels are not correlated, the systematic error and the random error of the dynamic star-spot can be derived as:

(15) $$\sigma_{x,2}^{2}={\left[\overset{n}{\underset{k=1}{\text{∑}}}\frac{\partial \bar{x}}{\partial x_{k}}\sigma_{x_{k}}\right]}^{2}={\left[\overset{n}{\underset{k=1}{\text{∑}}}\frac{I_{k}}{I_{0}}\sigma_{x_{k}}\right]}^{2}$$

(16) $$\sigma_{x,3}^{2}=\overset{n}{\underset{k=1}{\text{∑}}}\left[{\left(\frac{\partial \bar{x}}{\partial I_{k}}\right)}^{2}\sigma_{I_{k}}^{2}\right]=\overset{n}{\underset{k=1}{\text{∑}}}\left[{\left(\frac{x_{k}-\bar{x}}{I_{0}}\right)}^{2}\sigma_{I_{k}}^{2}\right]$$

where *σ_x_*_,2_ is the systematic error resulting from the use of the geometrical center instead of the energy center, *σ_x_*_,3_ is the random error caused by the image sensor noises, and *I*_0_ is the sum of all sampling values in centroiding window.

The energy center of the *k*th pixel can be calculated as:

(17) $$g_{x_{k}}=\frac{\iint_{C_{k}}xS(x,y)\text{dxdy}}{\iint_{C_{k}}S(x,y)\text{dxdy}}$$

where *C_k_* is the region of the *k*th pixel, *S*(*x*, *y*) is the dynamic star-spot imaging function, so the systematic error is written as:

(18) $$\begin{array}{c} \sigma_{x,2}^{2}={\left[\overset{n}{\underset{k=1}{\text{∑}}}\left(\frac{I_{k}}{I_{0}})(x_{k}-\frac{\iint_{C_{k}}xS(x,y)\text{dxdy}}{\iint_{C_{k}}S(x,y)\text{dxdy}}\right)\right]}^{2}\\ \sigma_{y,2}^{2}={\left[\overset{n}{\underset{k=1}{\text{∑}}}\left(\frac{I_{k}}{I_{0}})(y_{k}-\frac{\iint_{C_{k}}yS(x,y)\text{dxdy}}{\iint_{C_{k}}S(x,y)\text{dxdy}}\right)\right]}^{2} \end{array}$$

Substituting Equation (14) into Equation (16) yield, the random error of the *x̄* can be expressed as:

(19) $$\begin{array}{ll} \sigma_{x,3}^{2} & =\overset{\text{n}}{\underset{k=1}{\text{∑}}}\left[{\left(\frac{x_{k}-\bar{x}}{I_{0}}\right)}^{2}(\lambda^{2}(n_{DC}^{2}+n_{S,k}^{2}+n_{\text{PRNU},k}^{2}+n_{TN}^{2}+n_{\text{FPN}}^{2}+{n^{2}}_{\text{ADC}}))\right]\\ & =\overset{\text{n}}{\underset{k=1}{\text{∑}}}\left[{\left(\frac{x_{k}-\bar{x}}{I_{0}}\right)}^{2}(\lambda^{2}(n_{DC}^{2}+n_{RN}^{2}+n_{\text{FPN}}^{2}+{n^{2}}_{\text{ADC}})+\lambda I_{k}+\sigma_{\text{PRNU}}^{2}I_{k}^{2})\right]\\ & =\frac{\lambda^{2}(n_{DC}^{2}+n_{RN}^{2}+n_{\text{FPN}}^{2}+{n^{2}}_{\text{ADC}})}{I_{0}^{2}}\overset{n}{\underset{k=1}{\text{∑}}}{\left(x_{k}-\bar{x}\right)}^{2}\\ & +\frac{1}{I_{0}^{2}}\overset{n}{\underset{k=1}{\text{∑}}}[{\left(x_{k}-\bar{x}\right)}^{2}(\lambda I_{k}+\sigma_{\text{PRNU}}^{2}I_{k}^{2})] \end{array}$$

**Figure 5.**
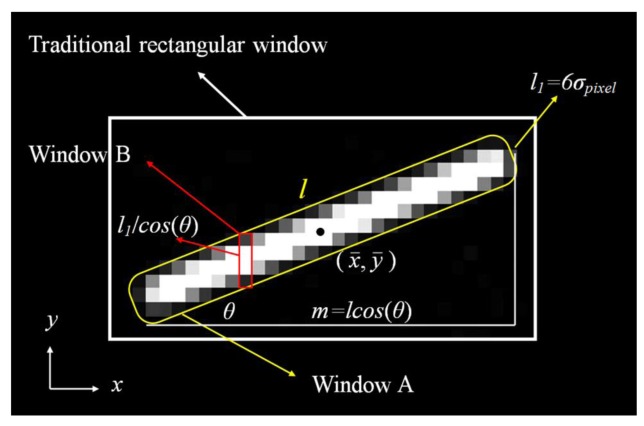
Detailed description of centroiding window.

The random error *σ_x_*_,3_ must be determined computing all the pixels with their noises in the centroiding window. If the centroiding window is set as the traditional rectangular window, the *σ_x_*_,3_ will obviously increase because many unnecessary pixels with fixed noises are computed. Generally, the centroiding window for the dynamic star-spot is set as the Window A in Figure 5. The length of Window A is the same as the star-spot trail, and its width is set to 6*σ_pixel_* pixels because the section plane of the star-spot trail fits Gaussian distribution. Dividing the Window A into many sub-windows like Window B whose width is just a pixel, some approximate expressions for calculating the *σ_x_*_,3_ are obtained as follows:

(20) $$\begin{array}{ll} \overset{n}{\underset{k=1}{\text{∑}}}{\left(x_{k}-\bar{x}\right)}^{2} & \approx 2\frac{l_{1}}{\cos(\theta )}\left[{\left(\frac{m}{2}\right)}^{2}+{\left(\frac{m}{2}-1\right)}^{2}+{\left(\frac{m}{2}-2\right)}^{2}\cdots \cdots +2^{2}+1^{2}\right]\\ & \approx 2\frac{6\sigma_{\text{pixel}}}{\cos(\theta )}\frac{{\left(m+1\right)}^{3}}{12}=\frac{\sigma_{\text{pixel}}{\left(l\cos(\theta )+1\right)}^{3}}{\cos(\theta )} \end{array}$$

where *l* is the length of the star-spot trail, *θ* is the angle between the star-spot trail and *x* axis, *l*_1_ is the width of centroiding window, *m* is the length of the star-spot trail in the *x* direction. As shown in Figure 5, the number of the pixels in the sub-window is *l_1_*/cos(*θ*),and the *m* is *l*cos(*θ*) pixels.

Obviously, the *m* is also the number of the sub-windows, and the total sampling value *I_B_* of the sub-window is *I*_0_/*m*:

(21) $$\begin{array}{ll} \overset{n}{\underset{k=1}{\text{∑}}}{\left(x_{k}-\bar{x}\right)}^{2}I_{k} & \approx 2\frac{I_{0}}{m}\left[{\left(\frac{m}{2}\right)}^{2}+{\left(\frac{m}{2}-1\right)}^{2}+{\left(\frac{m}{2}-2\right)}^{2}\cdots \cdots +2^{2}+1^{2}\right]\\ & \approx 2\frac{I_{0}}{m}\frac{{\left(m+1\right)}^{3}}{12}=\frac{I_{0}{\left(l\cos(\theta )+1\right)}^{3}}{6l\cos(\theta )} \end{array}$$

According to the dynamic star-spot model, the signal photoelectrons distribution of the sub-window fit approximately the Gaussian distribution with a Gaussian radius *σ_pixel_*/cos (*θ*). The sum of *I_k_*^2^ in the sub-window is approximately *I_B_*^2^/(3.6*σ_pixel_* /cos (*θ*)) statistically:

(22) $$\begin{array}{ll} \overset{n}{\underset{k=1}{\text{∑}}}{\left(x_{k}-\bar{x}\right)}^{2}I_{k}^{2} & \approx 2\frac{I_{B}^{2}\cos(\theta )}{3.6\sigma_{\text{pixel}}}\left[{\left(\frac{m}{2}\right)}^{2}+{\left(\frac{m}{2}-1\right)}^{2}+{\left(\frac{m}{2}-2\right)}^{2}\cdots \cdots +2^{2}+1^{2}\right]\\ & \approx 2\frac{I_{B}^{2}\cos(\theta )}{3.6\sigma_{\text{pixel}}}\frac{{\left(m+1\right)}^{3}}{12}=\frac{I_{0}^{2}{\left(l\cos(\theta )+1\right)}^{3}}{21.6\sigma_{\text{pixel}}l^{2}\cos(\theta )} \end{array}$$

Substituting Equations (20), (21) and (22) into Equation (19), we get the explicit expression of the random error for the high dynamic star-spot:

(23) $$\begin{array}{ll} \sigma_{x,3}^{2} & \approx \frac{\lambda^{2}(n_{DC}^{2}+n_{TN}^{2}+n_{\text{FPN}}^{2}+{n^{2}}_{\text{ADC}})\sigma_{\text{pixel}}{\left(l\cos(\theta )+1\right)}^{3}}{\cos(\theta )I_{0}^{2}}\\ & +\frac{\lambda {\left(l\cos(\theta )+1\right)}^{3}}{6I_{0}l\cos(\theta )}+\frac{\sigma_{\text{PRNU}}^{2}{\left(l\cos(\theta )+1\right)}^{3}}{21.6\sigma_{\text{pixel}}l^{2}\cos(\theta )}\\ \sigma_{y,3}^{2} & \approx \frac{\lambda^{2}(n_{DC}^{2}+n_{TN}^{2}+n_{\text{FPN}}^{2}+{n^{2}}_{\text{ADC}})\sigma_{\text{pixel}}{\left(l\sin(\theta )+1\right)}^{3}}{\sin(\theta )I_{0}^{2}}\\ & +\frac{\lambda {\left(l\sin(\theta )+1\right)}^{3}}{6I_{0}l\sin(\theta )}+\frac{\sigma_{\text{PRNU}}^{2}{\left(l\sin(\theta )+1\right)}^{3}}{21.6\sigma_{\text{pixel}}l^{2}\sin(\theta )} \end{array}$$

The *l, I*_0_ and *θ* are three main factors affecting the random error, the *l* and *I*_0_ are directly related to the exposure time, and the *θ* is decided by the direction of angular velocity. The total systematic error and the total random error can be written as:

(24) $$\begin{array}{cc} \sigma_{2} & =\sqrt{\sigma_{x,2}^{2}+\sigma_{y,2}^{2}}\\ \sigma_{3} & =\sqrt{\sigma_{x,3}^{2}+\sigma_{y,3}^{2}} \end{array}$$

**Figure 6.**
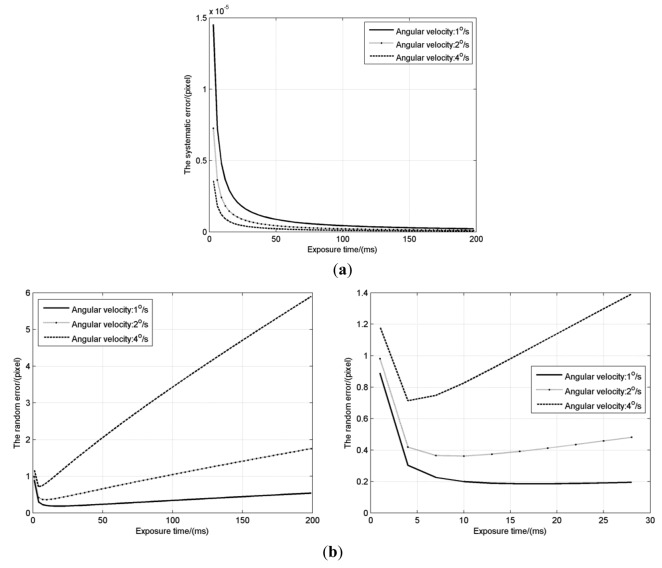
(**a**) The systematic error *σ*_2_ with different exposure times; (**b**) The random error *σ*_3_ with different exposure times, the right figure is the enlarged view in the range of 0 to 30 ms.

The simulation is carried out to obtain the two errors with different exposure times (*T_e_* = 0∼200 ms) and different angular velocities (*w* = 1°/s, 2°/s and 4°/s), with other parameters listed in Table 3. Effects of exposure time on the *σ*_2_, *σ*_3_ when the angle *θ* is 45°are shown in Figure 6.

Under highly dynamic conditions, the systematic error also changes like the S-curve error under static conditions. The amplitude of the systematic error, as shown in Figure 6a, decreases like an inverse proportion function with the increase of exposure time. This is because the *I*_k_/*I*_0_ is decreasing and the energy center of each pixel becomes nearer to the geometric center with the lengthening of the star-spot trail. The amplitude of the systematic error in the static condition is exp[−2(πσ)^2^]/π, which mainly depends on the Gaussian radius [9]. For the 0.7 pixel Gaussian radius, it is just about 2 × 10^−5^ pixels which is in agreement with the above simulation. Compared with the random error in Figure 6b, the systematic error is very small and negligible, so that the star location error under highly dynamic conditions mainly depends on the random error caused by the image sensor noises.

For the random error, it decreases slightly at first when the exposure time is very short, and then keeps rising at an almost fixed speed, which means an inflexion exists. According to Equation (23), at a given angular velocity, the random error is affected mainly by two factors: the total energy *I_0_* and the length *l* of the star-spot trail. When the exposure time is very short, the star-spot trail is very short and the total energy of each star-spot is very little. The increase of the exposure time means the gathering of the energy and the rising of the SNR, which results in that the random error decreases slightly at first. However, as the *I_0_* and *l* increase to some extent, the cube of the (*l*cos(*θ*) + 1) increases faster than the square of the *I*_0_, and the derivative of the random error to the exposure time is also almost a positive constant, so the random error begins to rise at a fixed speed. It is obvious that higher the angular velocity is, faster the length *l* increases, higher the speed is. We can denote the exposure time at the inflection point as *T_SLE_*, where the star tracker can achieve the highest star location accuracy.

## 4. Exposure Time Optimization

As an attitude measurement device, the main purpose of the star tracker is to provide highly-precision attitude information. Attitude accuracy is the most primary factor for performance evaluation. It can be estimated using the following equation [12]:

(24) $$E_{\text{cross}-\text{angle}}=\frac{A\cdot E_{\text{centroid}}}{N_{\text{pixel}}\cdot \sqrt{N_{\text{star}}}}$$

where *E_cross-angle_* is the estimation of the attitude error, *A* is the FOV of star tracker, *E_centroid_* is the average star location error, *N_pixel_* is the pixel array of the image sensor, and *N_star_* is the number of the star-spots in the star image. For a given star tracker, *A* and *N_pixel_* can be regarded as constants, so the attitude accuracy is mainly determined by *E_centroid_* and *N_star_* which are directly related to the exposure time. Hence, we can optimize the exposure time using the highest attitude accuracy as the criterion.

**Figure 7.**
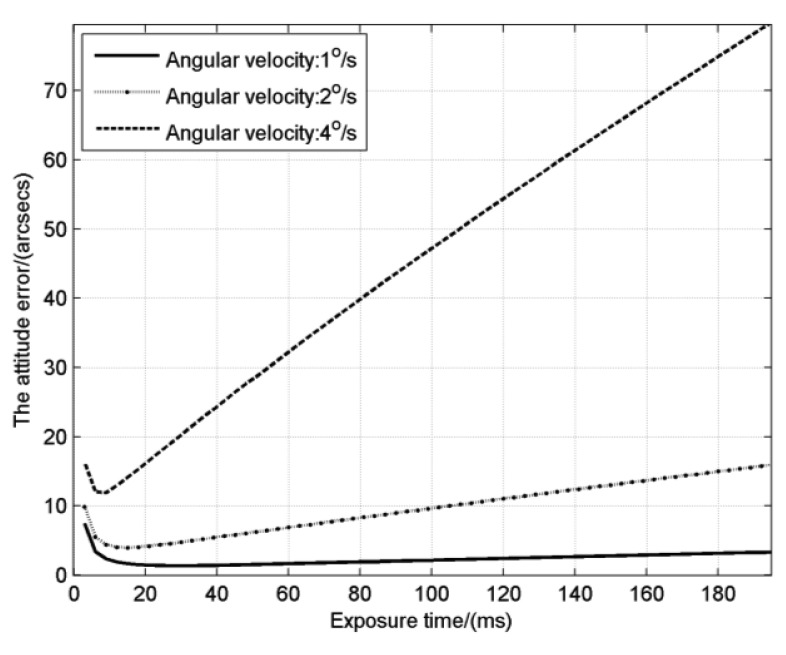
The attitude error with different exposure times.

Combining star detection sensitivity and star location error, effect of exposure time on the attitude error is shown in Figure 7. Variation regularity of the attitude error is basically consistent with the star location error. It decreases at first and then increases. The curve also has an inflection point which is just behind the inflection point (*T_SLE_*) of the star location error. According to Equation (24), the attitude error is directly proportional to *E_centroid_* and inversely proportional to the square root of *N_star_*. According to the previous analysis, when the exposure time is short, *N_star_* will increase and *E_centroid_* will reduce, so the attitude error decreases slightly at first. Then *E_centroid_* starts to rise when the exposure time pass *T_SLE_*, but the square root of *N_star_* increases more quickly than *E_centroid_*, which leads to the still decreasing of the attitude error. But with the continually increasing of exposure time, *E_centroid_* will get ahead of the square root of *N_star_* and the attitude error will increase. Finally, *N_star_* remains a constant and the increase speed of the attitude error is the same as the star location error.

**Figure 8.**
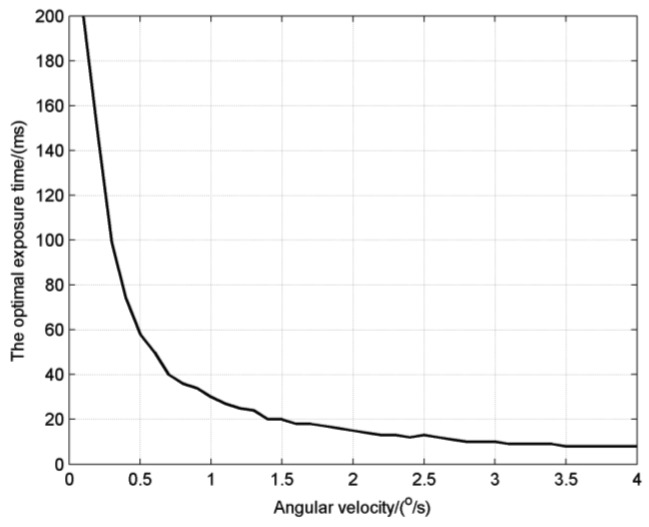
The optimal exposure times at different angular velocities.

The exposure time for the inflection point of the attitude error, denoted as the optimal exposure time, is between *T_SLE_* and *T_SDS_*, which reflects exactly the overall consideration on star detection sensitivity and star location error. It is noticed that when the angular velocity is relatively low, for example the 1°/s in the figure, the attitude error has a flat variation which means that we can choose a proper exposure time from a wide range, but for high angular velocities, we must set the exposure time carefully to obtain the best performance. The optimal exposure times at different angular velocities are calculated as shown in Figure 8. The result shows the optimal exposure time decreases like the inverse proportion function with the increase of angular velocity.

## 5. Night Sky Experiments

**Figure 9.**
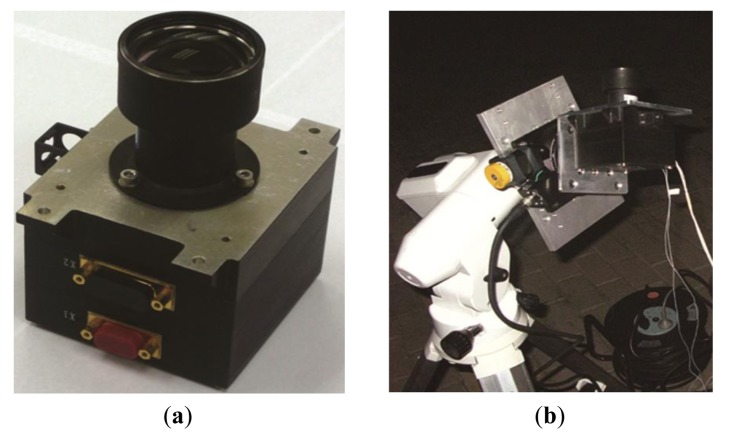
(**a**) The star tracker used in the experiment; (**b**) Night sky experiment setup.

**Figure 10.**
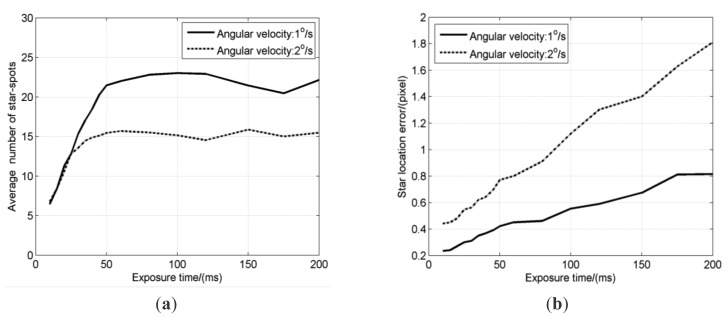
(**a**) The average number of the star-spots; (**b**) The average star location error with different exposure times in the experiment.

To validate the result of the simulations, night sky experiments using a recently developed star tracker were carried out. The star tracker, shown in Figure 9a, has a 14° × 14° rectangular FOV with a CMOS image sensor consisting of 2048 × 2048 pixels, with other parameters as listed in Table 3. The night sky experiment was performed at the Xinglong Astronomical Observatory of the Chinese Academy of Sciences. The star tracker was placed on a one dimension rotary table which was mounted on a tripod as shown in Figure 9b. Angular velocities of the rotary table were set to 1°/s and 2°/s. The exposure times were set to 10, 15, 20, 25, 30, 35, 40, 45, 50, 60, 80, 100, 120, 150, 175 and 200 ms respectively. For each of the 32 conditions, star-spot data of more than 100 star images were collected with the tested star tracker. The average number of the star-spots and the average star location error in each of conditions are calculated as shown in Figure 10.

In Figure 10a, the general regularity of the average number of star-spots is that it increases fast at first and then levels off slowly with the increasing exposure time. It can be seen from the figure that for the angular velocity of 1°/s, the average star number is 6.46 at the exposure time of 10 ms, then reaches slowly to the limit value 23.01 at 100 ms. For the angular velocity of 2°/s, it is 6.78 at 10 ms, reaches the limit value 15.69 at 60 ms. To sum up, the numerical value and general regularity of the average number of star-spots in the experiment are in agreement with the results of the previous simulations.

The average star location error, as shown in Figure 10b, keeps rising in the range of the exposure time 10 ms to 200 ms. For the angular velocity of 1°/s, the star location error is 0.24 pixels at 10 ms, and then increases steadily at a fixed speed and reaches 0.82 pixels at 200 ms. For the angular velocity of 2°/s, it is 0.44 pixel at 10 ms, reaches 1.81 pixel at 200 ms. The experimental results of star location error also agree basically with the simulations.

**Figure 11.**
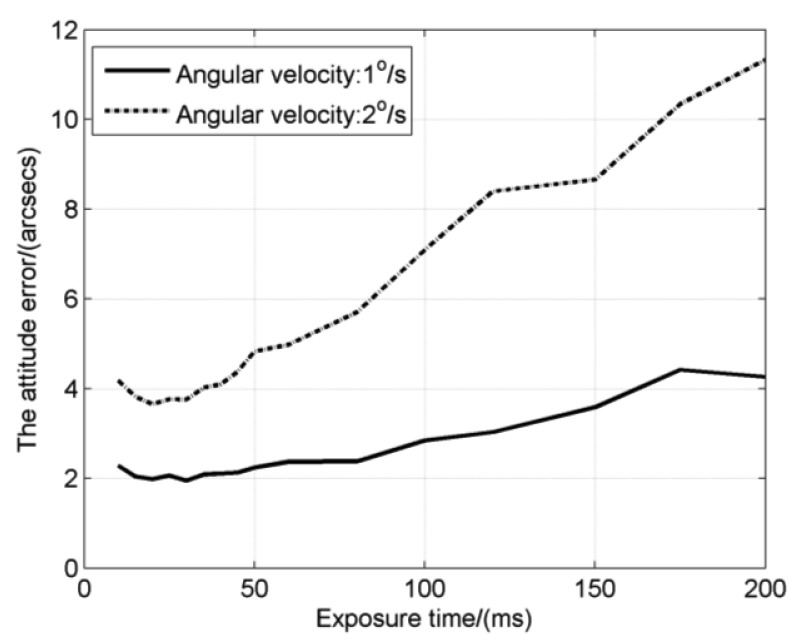
The attitude error with different exposure times in the experiment.

Finally, the attitude error is calculated as shown in Figure 11. The attitude error at angular velocities of 1°/s and 2°/s both have their inflection points, which are the corresponding optimal exposure times with the value of about 30 ms and 20 ms respectively. In previous simulations as shown in Figure 8, the optimal exposure times are 30.7 ms and 17.6 ms for 1°/s and 2°/s respectively, which are very close to the experimental results.

## 6. Conclusions

Effects of exposure time on star detection sensitivity and star location accuracy for a star tracker under highly dynamic conditions are analyzed in this paper. On this basis, the effects of exposure time on attitude accuracy and the corresponding optimal exposure times are also obtained. Some simulations and night sky experiments are carried out to validate these effects. The above content can be summarized as follows:

(1) Under highly dynamic conditions, star-spots will move across many pixels and form an obvious trail on the image sensor during the exposure time. The length of the star-spot trail is decided by the angular velocity and the exposure time, and the higher the angular velocity is, the larger the exposure time is, so the longer the trail is. This phenomenon has a bad influence on star detection sensitivity and star location accuracy, which result in low star tracker attitude accuracy.
(2) Firstly, the model of star detection sensitivity for the high dynamic condition is developed. According to the simulation, the star detection sensitivity rises rapidly at first with the increasing exposure time, and then slowly approaches a limit value. The limit values at different angular velocities are different, and the higher the angular velocity is, the smaller the limit value is. The critical exposure time *T_SDS_* for the limit value are also different, and the higher the angular velocity is, the shorter the *T_SDS_* is.
(3) Then this paper establishes the model of star location error for the highly dynamic conditions, and identifies the main error sources through theoretical analysis and simulation. The simulation result shows that when the exposure time is very short, the star-spot trail is short and negligible. Increasing of exposure time means the gathering of the energy, which leads to the rising of SNR and the decreasing of star location error. When the exposure time increases to some extent, the star-spot trail gradually becomes obvious and the star location error starts to rise at a steady speed, and the higher the angular velocity is, the larger the rise speed is. Overall, the star location error has a minimum at the inflection point with the exposure time *T_SLE_*.
(4) The estimation of attitude accuracy for highly dynamic conditions is obtained, which is used to determine the optimal exposure time of different angular velocities. The result shows the optimal exposure time is between the *T_SLE_* and the *T_SDS_*, and decreases like an inversely proportional function with the increase of angular velocity.
(5) Night sky experiments using a CMOS star tracker have been carried out, and the experimental results validate those of the simulations.

## Figures and Tables

**Table 1.** Main noises of CMOS image sensor.

**Noise**	**Formula**
Dark current noise	$n_{DC}=\sqrt{I_{\text{dark}}T_{e}}$
Photon shot noise	$n_{S}=\sqrt{S}$
Photon non-uniformity response noise	$n_{\text{PRNU}}=\sigma_{\text{PRNU}}S$
Quantization noise	$n_{\text{ADC}}=\frac{N_{\text{well}}}{2^{q}\sqrt{12}}$
Fixed pattern noise	$n_{\text{FPN}}=N_{\text{FPN}}$
Temporal noise	$n_{TN}=N_{TN}$

**Table 2.** *η* values with different lengths of the star-spot trail.

**Trail length *l*/pixel**	***η* value**	**Trail length *l*/pixel**	***η* value**
0	0.0831	10	0.0264
1	0.0804	11	0.0240
2	0.0740	12	0.0220
3	0.0651	13	0.0203
4	0.0547	14	0.0188
5	0.0450	15	0.0176
6	0.0401	16	0.0165
7	0.0347	17	0.0155
8	0.0321	18	0.0147
9	0.0295	19	0.0138

**Table 3.** Simulation parameters of star tracker with a CMV4000 CMOS image sensor.

**Parameter**	**Value**	**Parameter**	**Value**
Focal length	44.1637 mm	Pixel array	2048 × 2048
Optics aperture	40 mm	Principal point	(1094.4, 1078.0)
Gaussian radius	0.7 pixel	Pixel size	5.5 μm × 5.5 μm
Optical transmittance	0.85	Dark current	125 e/s
Atmospheric transmissivity	0.9	Photon non-uniformity	0.01
Quantum efficiency * Fill factor	0.6	Temporal noise	13 e
FOV	14° × 14°	Fixed pattern noise	35 e
Quantization bits	8	Full well charge	13,500 e
SNR threshold	5		
